# Intoxication of mammalian cells with binary clostridial enterotoxins is inhibited by the combination of pharmacological chaperone inhibitors

**DOI:** 10.1007/s00210-020-02029-3

**Published:** 2020-12-07

**Authors:** Katharina Ernst, Judith Sailer, Maria Braune, Holger Barth

**Affiliations:** Institute of Pharmacology and Toxicology, Ulm University Medical Center, 89081 Ulm, Germany

**Keywords:** Bacterial protein toxins, Pharmacological inhibitors, Chaperones, Cellular uptake, *Clostridioides difficile*, CDT toxin

## Abstract

Binary enterotoxins *Clostridioides difficile* CDT toxin, *Clostridium botulinum* C2 toxin, and *Clostridium perfringens* iota toxin consist of two separate protein components. The B-components facilitate receptor-mediated uptake into mammalian cells and form pores into endosomal membranes through which the enzymatic active A-components translocate into the cytosol. Here, the A-components ADP-ribosylate G-actin which leads to F-actin depolymerization followed by rounding of cells which causes clinical symptoms. The protein folding helper enzymes Hsp90, Hsp70, and peptidyl-prolyl *cis/trans* isomerases of the cyclophilin (Cyp) and FK506 binding protein (FKBP) families are required for translocation of A-components of CDT, C2, and iota toxins from endosomes to the cytosol. Here, we demonstrated that simultaneous inhibition of these folding helpers by specific pharmacological inhibitors protects mammalian, including human, cells from intoxication with CDT, C2, and iota toxins, and that the inhibitor combination displayed an enhanced effect compared to application of the individual inhibitors. Moreover, combination of inhibitors allowed a concentration reduction of the individual compounds as well as decreasing of the incubation time with inhibitors to achieve a protective effect. These results potentially have implications for possible future therapeutic applications to relieve clinical symptoms caused by bacterial toxins that depend on Hsp90, Hsp70, Cyps, and FKBPs for their membrane translocation into the cytosol of target cells.

**Supplementary Information:**

The online version contains supplementary material available at 10.1007/s00210-020-02029-3.

## Introduction

*Clostridioides difficile* toxin CDT, *Clostridium botulinum* C2 toxin, and *Clostridium perfringens* iota toxin are members of the family of binary actin-ADP-ribosylating toxins that cause enterotoxicity in humans and animals (Ohishi 1983; Songer 1996; Papatheodorou et al. 2018). These protein toxins consist of two separate proteins that are secreted by the bacteria and share a widely common cellular uptake mechanism and mode of action. The binding/translocation (B) component mediates the transport of the enzymatically active (A) component into the cytosol of target cells. Here, the A-component mono-ADP-ribosylates G-actin (Aktories et al. 1986; Schering et al. 1988; Popoff et al. 1988). This leads to depolymerization and destruction of actin filaments and causes rounding of adherent cells (Reuner et al. 1987; Wegner and Aktories 1988; Aktories and Wegner 1992). In vivo, rounding of epithelial cells in the intestine results in impairment of the gut barrier and thereby elicits clinical symptoms of enterotoxicity.

The C2 toxin is the prototype of this toxin family and its cellular uptake was studied in more detail. The B-component of C2 toxin C2II gets proteolytically activated (Barth et al. 2000). The resulting biologically active C2IIa forms heptameric complexes that bind to asparagine-linked carbohydrate structures that are located on the surface of all cell types (Ohishi et al. 1984; Eckhardt et al. 2000; Blöcker et al. 2000). C2I, the A-component, binds to C2IIa-heptamers and the complex is taken up into cells by receptor-mediated endocytosis. Vesicular ATPases cause acidification of the toxin-loaded endosomes. This leads to conformational changes of both components: the C2IIa-heptamer forms a translocation pore into the endosomal membrane and C2I is partially unfolded to translocate through the narrow pore into the cytosol of target cells (Barth et al. 2000; Haug et al. 2003b; Schleberger et al. 2006).

Cellular uptake and mode of action of the iota toxin and CDT are widely comparable and show some differences to C2 toxin. The B-components Ib and CDTb, respectively, facilitate the transport of the A-components Ia and CDTa into the cytosol ((Stiles and Wilkins 1986; Perelle et al. 1997) for review see (Barth and Stiles 2008; Barth and Ernst 2016)). Iota toxin and CDT are closely related and form the sub-group of iota-like toxins within the binary ADP-ribosylating toxins. They both bind to the lipolysis-stimulated lipoprotein receptor (LSR) and use CD44 as a co-receptor for cellular uptake (Papatheodorou et al. 2011; Wigelsworth et al. 2012).

During the last years, we showed that C2, iota, and CDT toxins require the assistance of folding helper enzymes for translocation of their A-components from endosomes to the cytosol (Barth and Ernst 2016; Ernst et al. 2017b, a). The chaperones heat shock protein (Hsp) 90 and 70, as well as isoforms of cyclophilins (Cyps) and FK506-binding proteins (FKBPs) facilitate the translocation of partially unfolded C2I, Ia, and CDTa through the respective pores into the cytosol (Haug et al. 2003a, 2004; Kaiser et al. 2009, 2011, 2012; Ernst et al. 2015, 2016, 2017a). Cyps and FKBPs are peptidyl-prolyl cis/trans isomerases (PPIases) that catalyze the rate-limiting step of cis/trans isomerization of prolyl-bond during protein folding (Schiene-Fischer 2014). Treatment of cells with specific pharmacological inhibitors blocks activity of folding helpers, which protects cells as well as more complex models like human intestinal organoids from intoxication with clostridial binary toxins. Radicicol (Rad) and VER-155008 (VER) inhibit activity of Hsp90 and Hsp70, respectively, by binding to their ATP-binding pockets. Cyclosporine A (CsA) and FK506 prevent activity of Cyps and FKBPs, respectively (Barth and Ernst 2016). Previously, we showed that combining all four inhibitors results in an enhanced protection of cells from intoxication with C2 toxin compared to application of the single inhibitors (Ernst et al. 2018b). Here, prompted by these earlier findings, we demonstrated that the inhibitor combination exhibits an enhanced inhibitory effect on CDT intoxication of cells. Since CDT contributes to the severe diseases caused by hyper-virulent CDT-expressing strains of *C. difficile* including the pseudomembranous colitis, the results can be a starting point for the development of novel pharmacological options to treat and/or prevent the diseases associated with CDT.

## Materials and methods

### Protein expression and purification

Protein toxin components were purified and activated as described before: C2I and C2IIa (Barth et al. 1998), CDTa and CDTb (Papatheodorou et al. 2010), Ia and Ib (Perelle et al. 1997).

### Cell culture

Cells were detached by trypsin and reseeded every 2–3 days for no more than 25 times. Incubation of cells occurred at 37 °C and 5% CO_2_ under humidified conditions. Vero cells (African green monkey kidney cells, DSMZ, Braunschweig, Germany) were cultured in MEM plus 10% heat-inactivated fetal calf serum (FCS) (GIBCO life technologies, Karlsruhe, Germany), 0.1 mM non-essential amino acids, 1 mM sodium pyruvate, 2 mM L-glutamine, and 100 U/mL of penicillin and 100 μg/mL of streptomycin. CaCo-2 cells (human epithelial colorectal adenocarcinoma cells, ATCC HTB-37, Manassas, VA, USA) were cultured in DMEM (GIBCO life technologies, Karlsruhe, Germany) plus 10% FCS, 1 mM sodium pyruvate, 0.1 mM non-essential amino acids, and 100 U/mL of penicillin and 100 μg/mL of streptomycin.

### Intoxication experiments

Cells were seeded into 24-well culture dishes. The following inhibitors of host cell chaperones were used: Rad (inhibitor of Hsp90), CsA (inhibitor of Cyps), and FK506 (inhibitor of FKBPs) were purchased from Sigma-Aldrich (Merck, Darmstadt, Germany), VER (inhibitor of Hsp70, Hsc70 and Grp78) was purchased from Tocris Bioscience (Wiesbaden-Nordenstadt, Germany). Bafilomycin A1 (BafA1, inhibitor of v-ATPase) was obtained from Calbiochem (Bad Soden, Germany). After pre-incubation of cells with inhibitors, toxin components were added. Images of cells were obtained using a Zeiss (Oberkochen, Germany) Axiovert 40CFL microscope with a Jenoptik (Jena, Germany) ProGres C10 CCD camera. Morphological changes induced by the toxins were analyzed by counting cells showing intoxication morphology (formation of protrusions, rounding of cells) and determining the percentage of intoxicated cells (Image J, National Institutes of Health, Bethesda, USA). Culture dishes and well plates were purchased from TPP Techno Plastic Products (Trasadingen, Switzerland).

### Analysis of ADP-ribosylation status of G-actin

CaCo-2 cells were pre-incubated with respective inhibitor combination and then intoxicated with CDT for given incubation periods. Cells were lysed in ADP-ribosylation buffer (1 mM DTT, 5 mM MgCl_2_ and 1 mM EDTA, 20 mM Tris-HCl pH 7.5 plus complete protease inhibitor (Roche, Mannheim, Germany)), followed by incubation with 100 ng CDTa and 10 μM biotin-labeled NAD^+^ (Trevigen, Gaithersburg, MD, USA) for 30 min at 37 °C for in vitro ADP-ribosylation of G-actin, which had not yet been ADP-ribosylated by CDTa during the previous incubation. Samples were subjected to SDS-PAGE, blotted, and ADP-ribosylated, i.e., biotin-labeled; G-actin was detected with streptavidin-peroxidase (Strep-POD, Sigma-Aldrich, Merck) using the ECL system. Equal amounts of protein loading were confirmed by ponceau S staining. Intensity of Western blot signals were quantified densitometrically and normalized to loading controls (ponceau S signals). Additionally, values were normalized to untreated control samples.

### TEER measurements

CaCo2 cells were seeded into 24-well hanging cell culture inserts (Millicell Cell Culture Inserts, EMD Millipore Corporation, Burlington, MA, USA). 1.1 × 10^5^ cells per filter were seeded. A dense monolayer was obtained after growing cells for 3–4 days. Fresh medium with or without inhibitors was added in a fresh 24-well plate and hanging inserts with cell monolayers were placed into the fresh medium. BafA1 is an inhibitor of vesicular ATPases and thereby also inhibits uptake of CDT, C2, and other toxins that escape from acidified endosomes. BafA1 was used as a positive control for inhibition of toxin uptake (Barth et al. 2000). After 30 min of pre-incubation at 37 °C, toxin components were added apically. TEER was measured every 30 min with the EVOM2 Voltohmmeter (World Precision Instruments, Friedberg, Germany). Raw data of resistance were transformed to unit area resistance by subtracting blank resistance and multiplying resulting data with effective surface area of used hanging insert membrane (here 0.3 cm^2^). Values were normalized to their respective starting value (*t*_0_).

### Cell viability

Vero cells were seeded into a 96-well plate. Cells were incubated with chaperone inhibitors at indicated concentrations for 6 h. Images were taken, then cells were washed to remove precipitation of inhibitors which occurred at higher concentrations. Then, MTS reagent (Promega, Mannheim, Germany) was added and after incubation for 1 h at 37 °C, absorbance at 490 nm was measured. Values were normalized to untreated control cells (control = 100%). Results from 4 independent experiments with triplicates are shown.

### Reproducibility of experiments

All experiments were performed independently at least two times. Results from representative experiments are shown in figures if not indicated otherwise.

## Results

### Combination of pharmacological inhibitors shows a more pronounced delay of CDT intoxication than application of the respective individual inhibitors

**Fig. 1:**
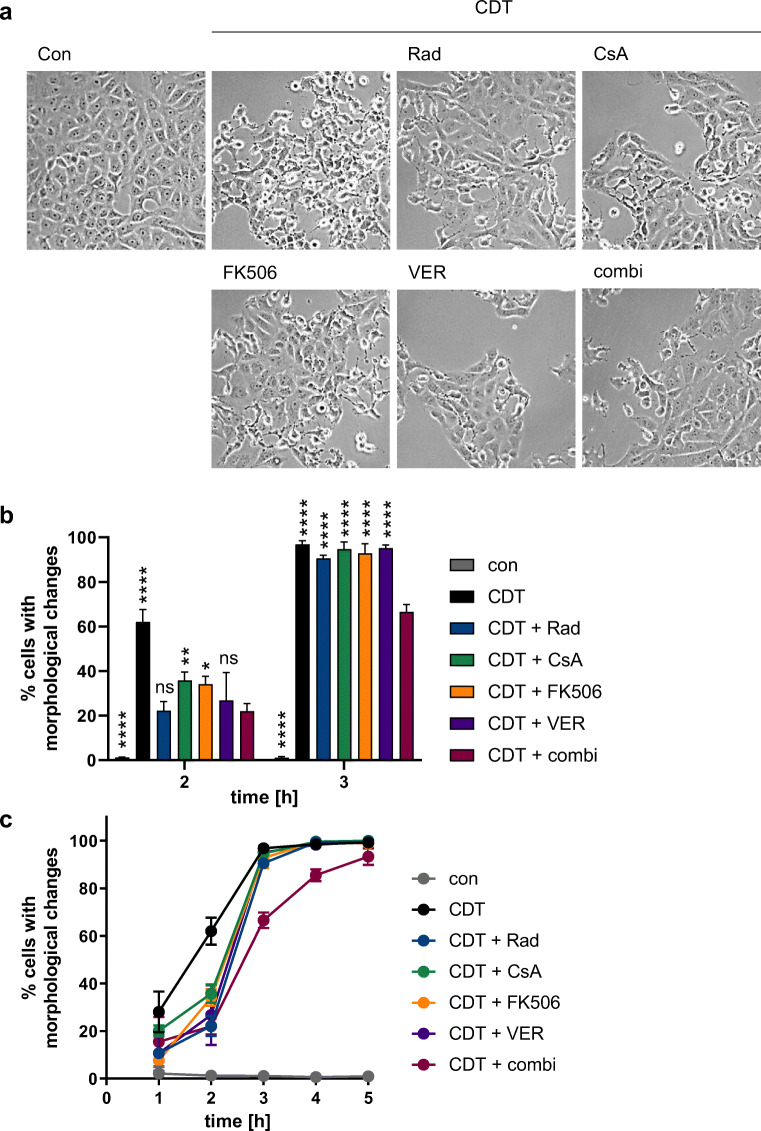
Combined pharmacological inhibition of Hsp90, Hsp70, Cyps, and FKBPs delays intoxication of Vero cells with CDT. Vero cells were pre-incubated at 37 °C for 30 min with the single inhibitors or a combination of the inhibitors (CsA 20 μM, FK506 20 μM, radicicol 20 μM and VER 30 μM). Cells were then challenged with 50 ng/mL CDTa + 100 ng/mL CDTb. For control, cells were left untreated or treated only with CDT. Cells were further incubated at 37 °C and images were taken at the indicated time points. **a** Images show the morphological changes induced by the toxin after 2 h of incubation. **b** Percentage of cells with morphological changes was determined from images at the indicated time points. Values are given as mean ± SD (*n* = 3). Significance was tested using two-way ANOVA followed by Dunnett’s multiple comparison test. (* *p* ≤ 0.05, ** *p* ≤ 0.01, **** *p* ≤ 0.0001, ns, not significant vs CDT + combi). **c** Time course of intoxication with CDT determined from images

Intoxication of adherent cells like Vero cells with CDT leads to specific morphological changes, i.e., rounding of cells. This is a direct effect of the mode of action of CDT and other actin-ADP-ribosylating toxins like C2 and iota toxin and is used as a robust and highly specific endpoint to determine the degree of intoxication. Images in Fig. 1a show that CDT caused rounding of cells after 2 h of intoxication. The percentage of cells showing this change in morphology was determined from the pictures (Fig. 1b). If cells were pre-incubated with the single chaperone and PPIase inhibitors, a delay in intoxication was observed after 2 h. A comparable effect was achieved if all four inhibitors were applied in combination. However, after longer incubation periods, only the combination of inhibitors and not the inhibitors alone caused a delay in intoxication (Figs. 1b, c). The solvents of the inhibitors had no effect on CDT intoxication (supplemental Fig. 1).

### Combined pharmacological inhibition of chaperones/PPIases delays intoxication with CDT in a human colon epithelial cell line

**Fig. 2:**
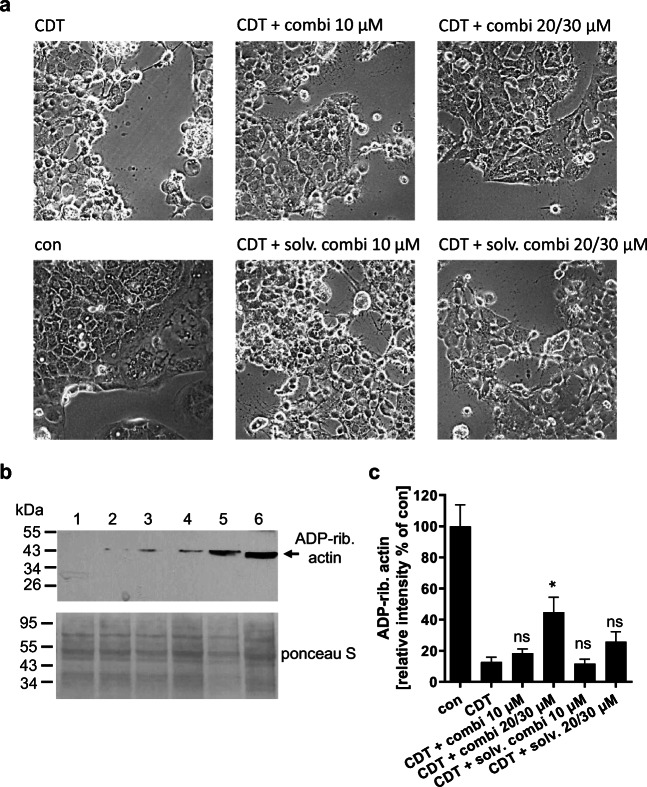
Combined inhibition of Hsp90, Hsp70, Cyps, and FKBPs delays intoxication of human CaCo-2 cells with CDT. CaCo-2 cells were pre-incubated at 37 °C for 30 min with the combination of the inhibitors in two different concentrations (10 μM each or 20 μM CsA/FK506/radicicol, 30 μM VER). Cells were then challenged with 150 ng/mL CDTa + 300 ng/mL CDTb. For control, cells were left untreated, treated only with CDT or with CDT in the presence of the respective solvent amount (ethanol or DMSO) of the respective inhibitor combinations. Cells were further incubated at 37 °C for 4.5 h. Subsequently, images were taken (**a**) and cells were lysed. (**b**) Cell lysates were incubated with fresh CDTa in the presence of biotin-labeled NAD^+^ allowing ADP-ribosylation of G-actin that has not been modified during the incubation of cells. Cell lysates were subjected to SDS-PAGE followed by Western blot analysis. Biotin-labeled G-actin was detected by Strep-POD and chemiluminescence. Transfer of protein by Western blotting was confirmed by ponceau S staining. Lane 1 = CDT, Lane 2 = CDT + solv. combi 10 μM, Lane 3 = CDT + combi 10 μM, Lane 4 = CDT + solv. combi 20/30 μM, Lane 5 = CDT + combi 20/30 μM, Lane 6 = con. (**c**) Western blot signals were quantified by densitometry from 3 independent experiments. Values were normalized to protein loading, i.e., the ponceau S signal of each sample and in each experiment, values were normalized to untreated controls. Values are given as mean ± SEM (*n* = 6, duplicates from 3 independent experiments). Significance was tested using one-way ANOVA followed by Dunnett’s multiple comparison test. (*p ≤ 0.05, ns, not significant vs CDT)

A delay in CDT intoxication by the inhibitor combination was also observed in the human colon carcinoma cell line CaCo-2 (Fig. 2a). Since the protective effect on cell morphology was not as clearly observable in CaCo-2 cells as in Vero cells, we analyzed the ADP-ribosylation status of G-actin in these cells to confirm the results. Therefore, cells were lysed and subsequently incubated with fresh CDTa plus biotin-labeled NAD^+^ in vitro. This leads to ADP-riboslyation of the portion of G-actin that has not been modified in the living cells before. The presence of biotin-labeled NAD^+^ results in biotin-labeling of that portion of G-actin which was detected by Western blot. Therefore, a strong signal in the blot indicates no modification of actin in the living cells, which was observed for untreated control samples (Fig. 2b). A weak signal means that most of the actin has been ADP-ribosylated by the toxin in the living cells thus, could not serve as a substrate in the in vitro ADP-ribosylation. In samples that have been treated with only CDT, no signal was detectable. Samples pre-treated with the lower concentration of the inhibitor combination prior to CDT intoxication showed a weak signal. However, this signal was comparable to samples treated with only solvent and CDT. A significantly increased signal was obtained in samples treated with the higher concentration of inhibitors and CDT, even in comparison to the respective solvent control. Figure 2b shows the result of one representative experiment. Since the protein loading of the samples slightly varied between the different treatments, a quantification of Western blot signals was performed with normalization of Western blot signal to the protein loading. Figure 2c comprises the results of 3 independent experiments and demonstrates a significant inhibitory effect of the combination of inhibitors in the higher concentration on the intoxication of CaCo-2 cells with CDT.

**Fig. 3:**
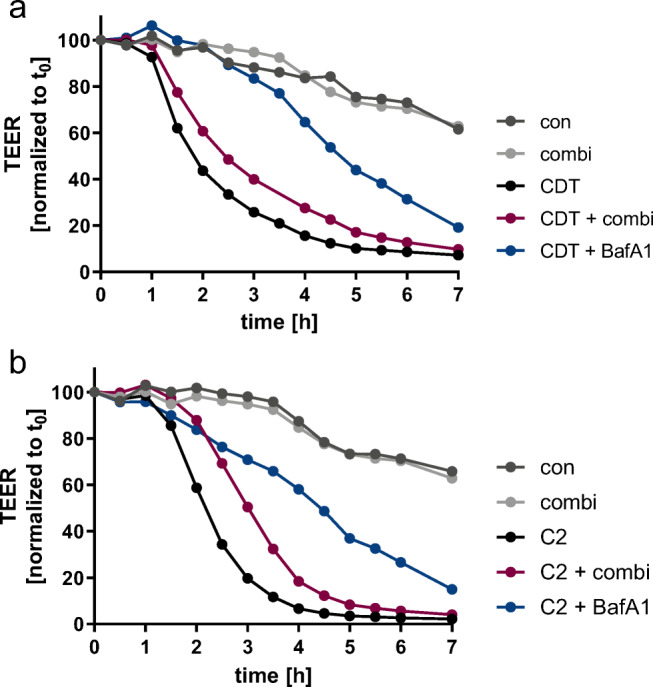
Combination of chaperone inhibitors delays impairment of epithelial integrity of CaCo-2 monolayers by CDT and C2 toxin. Caco-2 monolayers were treated with the combination of inhibitors or their respective solvents for 30 min. (**a**) CDT (30 ng/mL CDTa + 60 ng/mL CDTb) or (**b**) C2 (50 ng/mL C2I + 100 ng/mL C2IIa) were added and TEER was measured at indicated time points. For control, cells were left untreated or were treated only with the inhibitor combination. Values were normalized to t_0_ values (*t*_0_ = 100%). Single values of one representative time course out of three independent experiments are shown

TEER measurements were performed to analyze the epithelial integrity of confluently grown CaCo-2 monolayers after treatment with CDT or C2 toxin in the presence of the inhibitor combination (Fig. 3). Both, CDT and C2 toxin caused a strong reduction in TEER, which was delayed by the inhibitor combination. This inhibitory effect was more pronounced for C2 toxin in comparison with CDT. The established inhibitor BafA1, which inhibits acidification of endosomes, was used as a control and led to a delayed toxin-induced reduction of TEER. After 3–5 h of incubation, BafA1 alone led to a decrease in TEER values (supplemental Fig. 2). Solvents of the inhibitors had no inhibitory effect in this assay (not shown). Cells treated with the inhibitor combination alone behaved comparable to untreated controls.

### Delay of C2 and CDT intoxication by inhibitor combination allows to reduce the concentrations of the individual inhibitors

**Fig. 4:**
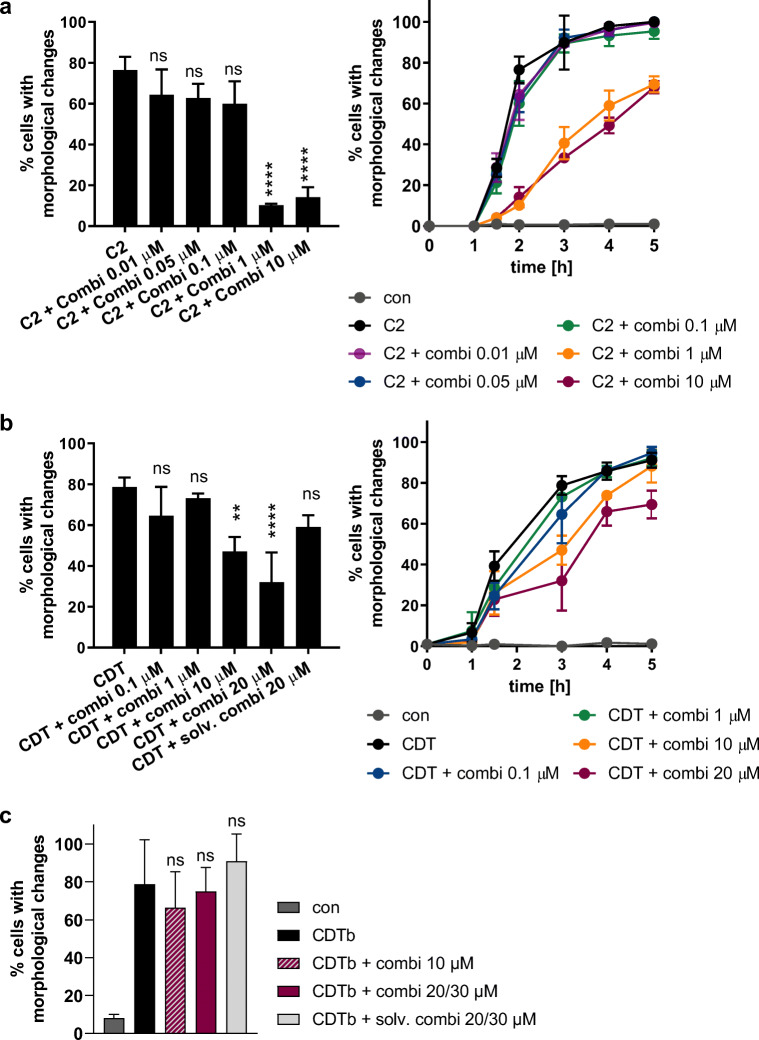
Reduced concentrations of inhibitors still delay the intoxication of cells with C2 toxin and CDT if applied in combination. Vero cells were pre-incubated with the combination of inhibitors in different concentrations. (**a**) C2 toxin (50 ng/mL + 100 ng/mL C2IIa) was added. Bar graph shows the percentage of cells with morphological changes determined from images taken after 2 h of C2 incubation. (**b**) CDT was added (50 ng/mL CDTa + 100 ng/mL CDTb). Bar graph shows the percentage of intoxicated cells after 3 h of CDT incubation. Values are given as mean ± SD (*n* = 3). Significance was tested using one-way ANOVA followed by Dunnett’s multiple comparison test. (**p ≤ 0.01, ****p ≤ 0.0001, ns, not significant vs CDT). (**c**) Vero cells were pre-incubated at 37 °C for 30 min with the combination of the inhibitors (10 μM each) or their corresponding solvents. For control, cells were left untreated. CDTb (600 ng/mL) was added and images were taken after 1 h. Percentage of cells with morphological changes, i.e., cell rounding were determined from images. Values are given as mean ± SD (*n* = 6). Significance was tested using one-way ANOVA followed by Dunnett’s multiple comparison test. (ns, not significant vs CDTb)

Since the combination of inhibitors alone impairs cell viability after longer incubation periods (> 24 h) (Ernst et al. 2018b), we tested whether the inhibitor combination is also effective if the concentration of each inhibitor is reduced. A clear delay in intoxication with C2 toxin was observed if inhibitors were applied in concentrations down to 1 μM (Fig. 4a). With the concentration series used in this experiment, a threshold was observed meaning that no inhibitory effect was detected when 0.1 μM of each inhibitor was applied. For CDT, a concentration-dependency was also observed (Fig. 4b). The lowest concentration of the inhibitors, for which a delay was seen, was 10 μM. Interestingly, the recently described cytotoxic effect of the binding/translocation component CDTb in the absence of its enzyme component CDTa was not affected by the combination of chaperone inhibitors (Fig. 4c).

**Fig. 5:**
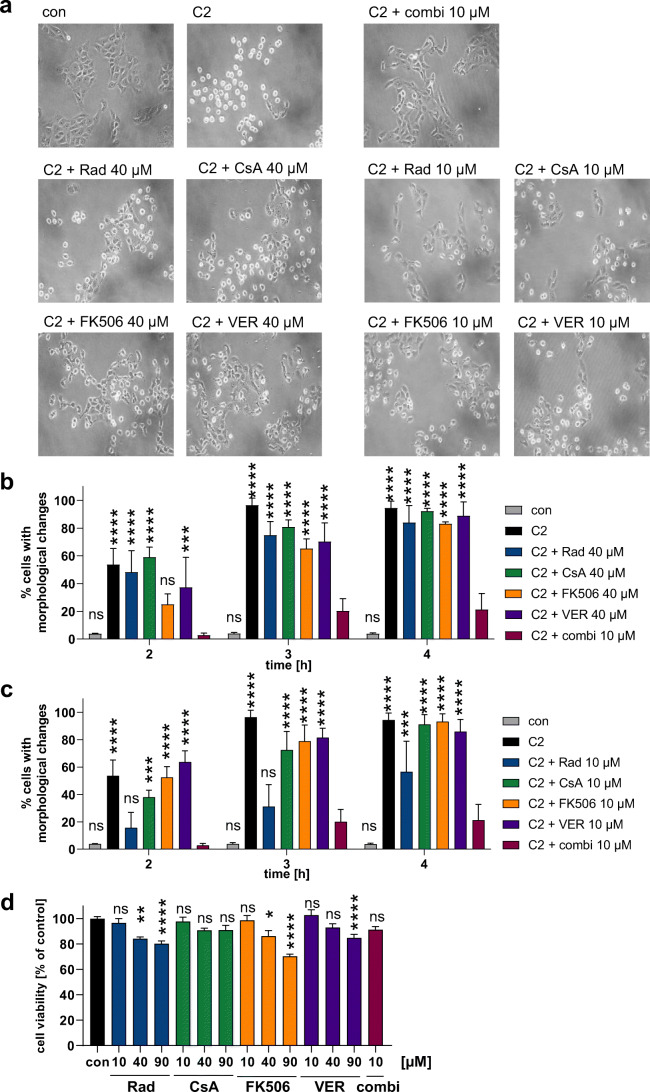
Inhibitor combination protects cells from C2 intoxication in reduced concentrations compared to single inhibitors. Vero cells were pre-incubated with single inhibitors (Rad, CsA, FK506, VER, 40 μM, or 10 μM) or with the combination of inhibitors (10 μM of each inhibitor). C2 toxin was added (50 ng/mL C2I + 100 ng/mL C2IIa) and cell morphology was monitored. (**a**) Cell images are shown exemplarily after 3 h of incubation with C2 toxin. Percentage of cells with morphological changes was determined from cell images. For better visualization, comparison of 40 μM of single inhibitors vs 10 μM of inhibitor combination are shown in (**b**) and comparison of 10 μM of single inhibitors vs 10 μM of inhibitor combination are shown in (**c**). Values for con, C2, and C2 + combi 10 μM are identical in both graphs. Values are given as mean ± SD (*n* = 3). Significance was tested using two-way ANOVA followed by Dunnett’s multiple comparison test. (****p ≤ 0.0001, ***p ≤ 0.001, ns, not significant vs C2 + combi 10 μM). (**d**) Vero cells were incubated with Rad, CsA, FK506, VER, or the combination of all four inhibitors at indicated concentrations for 6 h. Cell images were taken. Then, medium was exchanged to remove precipitation in the inhibitor samples and cell viability was measured by MTS assay. Values are given as mean ± SEM (*n* = 4 (triplicates from 4 independent experiments)). Significance was tested using one-way ANOVA followed by Dunnett’s multiple comparison test. (****p ≤ 0.0001, ***p ≤ 0.001, *p ≤ 0.05, ns, not significant vs con)

For C2 toxin, we also showed that treatment with lower concentrations of each inhibitor in combination (10 μM each = 10 + 10 + 10 + 10) still exhibits an enhanced protective effect when compared to a corresponding concentration of 40 μM of each single inhibitor (Fig. 5a, b). Moreover, 10 μM of inhibitor combination (10 μM each) protected cells better than application of single inhibitors at a concentration of 10 μM (Fig. 5c). The inhibitor combination was also superior when applied at lower concentrations (5 μM each) compared to 20 μM of each single inhibitor (supplemental Fig. 3). Comparing the effect of single inhibitors in concentrations that would correspond to the combination used before (20 + 20 + 20 + 30 = 90 μM) was not analyzed because inhibitors at 90 μM either impaired cell viability or were not soluble (Fig. 5d, supplemental Fig. 4).

**Fig. 6:**
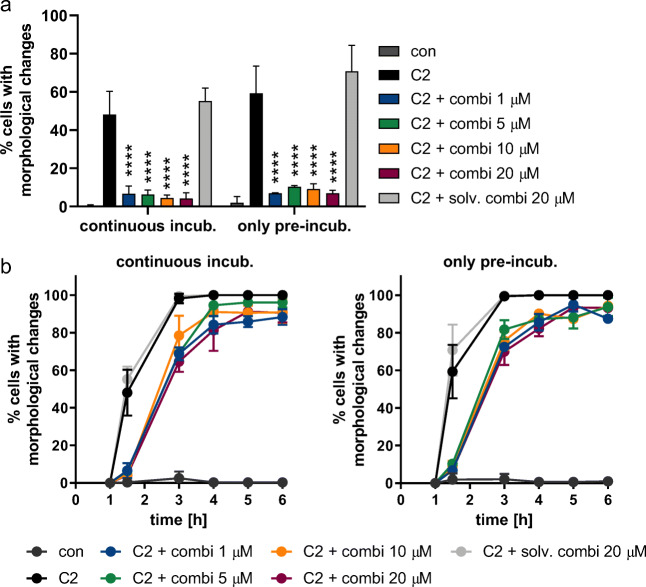
Pre-incubation of cells with inhibitor combination is sufficient for delaying C2 intoxication. Vero cells were pre-incubated with the inhibitor combination in different concentrations. One set of samples (left) was immediately treated with C2 toxin (50 ng/mL C2I + 100 ng/mL C2IIa), in the other set (right) the inhibitor-containing medium was removed, replaced by fresh medium without inhibitors and then, C2 toxin was added in the same concentration as in the other set. Percentage of intoxicated cells was determined from images. **a** Percentage of cells with morphological changes after 1.5 h of intoxication. Significance was tested using two-way ANOVA followed by Dunnett’s multiple comparison test. (****p ≤ 0.0001 vs CDT). **b** Time course of intoxication. Values are given as mean ± SD (*n* = 3)

Up to now, in every intoxication experiment, cells were pre-incubated with inhibitors for 30 min. Then, toxin was added with the inhibitors still present in the cell culture medium. Here, we showed that C2 intoxication was delayed even if inhibitors were removed after 30 min pre-incubation (Fig. 6). The observed inhibitory effect in samples with only pre-incubation was comparable to samples with a continuous inhibitor incubation.

### Inhibitory effect of inhibitor combination is most pronounced for C2 intoxication compared to CDT and iota intoxication of cells

**Fig. 7:**
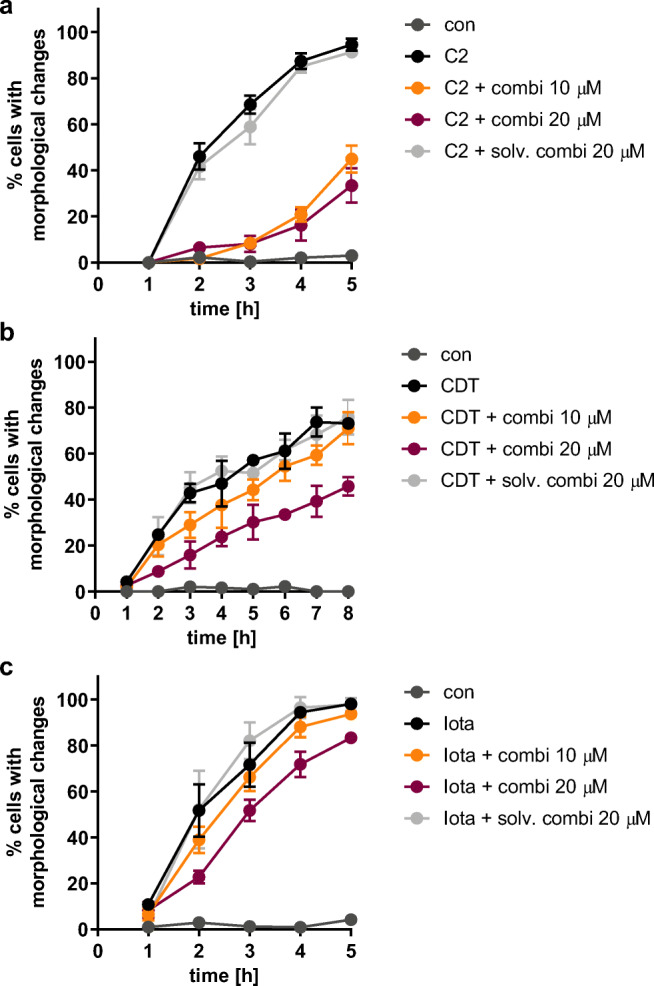
Protective effect of inhibitor combination is most pronounced for C2 intoxication of cells compared to CDT and iota intoxications. Vero cells were pre-incubated with inhibitor combinations (10 μM Rad, CsA, FK506, and VER or 20 μM Rad, CsA, FK506, and 30 μM VER). For control, cells were left untreated or treated with the amount of solvents that corresponds to the higher inhibitor concentrations (20/30 μM). Cells were then challenged with (**a**) 50 ng/mL C2I + 100 ng/mL C2IIa, (**b**) 35 ng/mL CDTa + 70 ng/mL CDTb, or (**c**) 25 ng/mL Ia + 50 ng/mL Ib. Percentage of cells with morphological changes was determined from images. Values are given as mean ± SD (*n* = 3)

The effect of the inhibitor combination in two different concentrations on C2, CDT, and iota intoxication of cells was tested. The time courses of intoxication in Fig. 7 show that the inhibitory effect was most obvious for C2 intoxication in both concentrations tested. A slight delaying effect of the lower inhibitor concentration and a clear effect of the higher concentration were observed for CDT intoxication. For the iota toxin, only the higher inhibitor concentration delayed the intoxication of cells.

## Discussion

Bacterial AB-type toxins are important virulence factors that cause severe diseases like cholera, whooping cough, or diphtheria. The clostridial C2 and iota toxin cause enterotoxicity in animals like calves and lambs and therefore pose a threat to livestock health and survival (Kurazono et al. 1987; Songer 1996). CDT harbors medical relevance in humans (Papatheodorou et al. 2018). *C. difficile* infections (CDI) present one of the most common healthcare-associated infections. CDI can elicit gastrointestinal symptoms ranging from diarrhea to pseudomembranous colitis and in most severe cases to toxic megacolon and sepsis. These symptoms are caused by the secreted AB-type toxins A (TcdA) and B (TcdB) (Papatheodorou et al. 2018). The emerging of hyper-virulent *C. difficile* strains aggravates the threat to patients and complicates the already difficult treatment (Gerding et al. 2014; Papatheodorou et al. 2018). Hyper-virulent *C. difficile* strains show increases in toxin secretion, antibiotic resistance, morbidity, and mortality as well as reoccurrence of symptoms. Moreover, these strains produce CDT as an additional toxin to TcdA and TcdB (Gerding et al. 2014). CDT ADP-ribosylates G-actin in target cells which leads to F-actin depolymerization and rounding of adherent cells. This further contributes to the impairment of the intestinal barrier which can be experimentally demonstrated by TEER measurements of epithelial cell layers. Additionally, CDT elicits the formation of microtubule-based protrusions in target cells (Schwan et al. 2009, 2011). Thereby, adherence of *C. difficile* to cells is enhanced in vitro and in vivo, and colonization of the gut is improved.

**Fig. 8:**
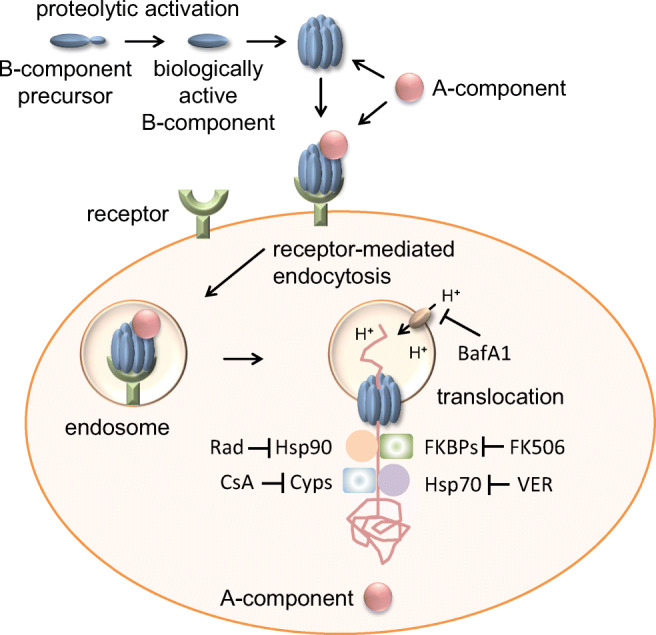
Uptake mechanism of clostridial binary toxins CDT, C2, and iota toxin. After proteolytic activation, the B-component forms heptamers and binds to a cell surface receptor. The A-component binds to the heptamer and the complex is taken up by receptor-mediated endocytosis. Acidification of the endosomal lumen leads to insertion of the B-heptamer into the endosomal membrane thereby forming a pore through which the partially unfolded A-component translocates into the cytosol. This translocation step is facilitated by cellular protein folding helper enzymes Hsp90, Hsp70, Cyps, and FKBPs. Inhibition of these host cell factors by specific pharmacological inhibitors protects cells from intoxication with CDT, C2, and iota toxins. Here, we show that combining these inhibitors has an enhanced protective effect on intoxication with clostridial binary toxins compared to application of individual inhibitors. BafA1, bafilomycin A1; FKBPs, FK506-binding proteins; Cyps, cyclophilins; Rad, radicicol; CsA, cyclosporine A; VER, VER-155008

Currently, *C. difficile* infection is treated with specific antibiotics. Moreover, an antibody against TcdB is available. Since the secreted toxins are the cause of disease, further therapeutic strategies are required that are targeted at the toxins. Pharmacological inhibition of chaperones and PPIases protects cells from intoxication with CDT, C2 and iota toxin, and several other toxins (Ernst et al. 2017b). We showed that CDT, C2, and iota toxin directly bind to Hsp90, Hsp/c 70, and different isoforms of Cyps (CypA, Cyp40) and FKBPs (FKBP51, FKBP52) (Kaiser et al. 2009, 2011, 2012; Ernst et al. 2015, 2016, 2017a). Table 1 gives an overview of toxins that are dependent or independent of Hsps or PPIases. Inhibitors of these chaperones/PPIases specifically inhibited the membrane translocation of the toxins’ enzyme components from endosomes to the cytosol (Fig. 8). Other steps of toxin uptake or mode of action like receptor-binding or in vitro enzyme activity were not affected by the inhibitors. Moreover, we recently showed that combining individual chaperone inhibitors has an enhanced protective effect against the intoxication with C2 toxin (Ernst et al. 2018b).

**Table 1:** Overview of toxins that are dependent or independent of Hsp90/Hsp70/PPIases

Enzyme activity	Toxin	Hsp90	Cyp	FKBP	Hsc70 Hsp70	References
Toxins that require Hsp90/Hsp70/PPIases						
ADP-RT	*C. botulinum* C2 toxin	✔	✔	✔	✔	Haug et al. (2003a); Kaiser et al. (2009, 2012); Ernst et al. (2015, 2017a)
	*C. perfingens* Iota toxin	✔	✔	✔	✔	Haug et al. (2004); Kaiser et al. (2011, 2012); Ernst et al. (2015, 2016, 2017a)
	*C. difficile* CDT toxin	✔	✔	✔	✔	Kaiser et al. (2011, 2012); Ernst et al. (2015, 2017a)
	*Photorhabdus luminescens* PTC3 toxin	✔	✔	✔	n.a.	Lang et al. (2014)
	*Corynebacterium diphtheriae* toxin	✔	✔	✔	✔	Ratts et al. (2003); Dmochewitz et al. (2011); Schuster et al. (2017)
	*Bordetella pertussis* toxin	✔	✔	n.a.	n.a.	Ernst et al. (2018a); Kellner et al. (2019)
	*Vibrio cholerae* toxin	✔	-	n.a.	✔	Taylor et al. (2010); Burress et al. (2014, 2019)
MP	Botulinum and tetanus neurotoxin	✔	-	n.a.	-	Azarnia Tehran et al. (2016)
MP	*Photobacterium damselae subsp. piscicida* AIP56	✔	✔	-	n.a.	Rodrigues et al. (2019)
Toxins that do not require Hsp90/Hsp70/PPIases						
GT	*C. difficile* TcdA, TcdB	-	-	-	n.a.	Haug et al. (2003a); Kaiser et al. (2009, 2012); Dmochewitz et al. (2011); Steinemann et al. (2018)
MP	*B. anthracis* lethal toxin	-	-	-	n.a.	Haug et al. (2003a); Zornetta et al. (2010); Dmochewitz et al. (2011)

✔ = folding helper is required for cellular uptake

*n.a.*, not analyzed; *PPIase*, peptidyl prolyl cis/trans isomerase; *ADP-RT*, ADP-ribosyltransferase; *MP*, metallo protease, *GT*, glycosyltransferase

In this study, we extended these findings and demonstrated that the combination of chaperone/PPIase inhibitors has an enhanced protective effect against the medically relevant CDT. This inhibitory effect was shown by analyzing CDT-induced changes in morphology, ADP-ribosylation status of actin in CaCo-2 cells, and transepithelial resistance of CaCo-2 monolayers. Although this enhanced protective effect was statistically significant, we observed that the inhibitor combination exerts a stronger protection against C2 toxin compared to CDT and iota toxin. C2, CDT, and iota share a similar structure and uptake mechanism. However, several differences between C2 and iota-like toxins that amongst others include iota toxin and CDT have been described. For example, iota toxin and CDT bind to the same receptor, the LSR (Papatheodorou et al. 2011, 2018), while C2 toxin binds to carbohydrate structures on the cell surface (Eckhardt et al. 2000). The enzyme component of C2 toxin translocates from early endosomes, the enzyme component of iota toxin from intermediate endosomes, and requires a membrane potential gradient (Gibert et al. 2007). Moreover, during the last years, it has been described that the binding/translocation components of iota toxin and CDT show a cytotoxic effect that is independent of their enzyme components (Nagahama et al. 2011; Fischer et al. 2018, 2020; Kronhardt et al. 2018; Korbmacher et al. 2020). We observed that high concentrations of CDTb or Ib (> 200–400 ng/mL) cause rapid cell rounding of Vero cells and decrease of cell viability in the absence of CDTa or Ia (Fischer et al. 2018, 2020; Korbmacher et al. 2020). Moreover, pore formation by CDTb in black lipid bilayers and living CaCo-2 cells was shown (Kronhardt et al. 2018; Korbmacher et al. 2020; Fischer et al. 2020). Here, we showed that the cytotoxic CDTb effect was not inhibited by the combination of the chaperone inhibitors, which might be one reason why the inhibitory effect of the inhibitor combination is weaker on CDT compared to C2 toxin.

Despite this weaker effect on CDT compared to C2 toxin, a delay in intoxication of ~ 2 h could still be relevant in context of clinical symptoms. Symptoms of CDI last for longer time periods than analyzed in this study (days vs hours). Nevertheless, extrapolation of toxin concentrations and time courses of intoxication from cell culture to the in vivo situation cannot be done one-to-one. The effectiveness of chaperone inhibitors as an anti-toxin strategy with protective effects has to be further investigated in an animal model, e.g., the intestinal loop model in mice (Fischer et al. 2020). To improve protective effects over longer time periods, i.e., days, a repetitive application of low-dosed inhibitors is also conceivable. Moreover, anti-toxin strategies are not supposed to replace but rather to support the existing therapeutic strategies. Thereby, the disease can be tackled on different levels: antibiotics to eliminate toxin-producing bacteria, toxin antibodies to neutralize “free” unbound toxin, and inhibitors (e.g., chaperone inhibitors) to protect cells from toxin molecules that have been internalized already.

Moreover, we showed that the concentration of the individual inhibitors could be reduced to achieve a protective effect against CDT and C2 toxin if inhibitors are applied in combination. In fact, the inhibitor combination still showed an enhanced protective effect against C2 toxin when compared to increased concentrations of the single inhibitors, i.e., 40 μM (or 20 μM) of single inhibitors vs 10 μM (or 5 μM) of each inhibitor in combination. For C2 toxin, pre-incubation of cells with the inhibitor combination was sufficient to protect cells from intoxication. These findings suggest that inhibitor concentrations and exposure times could also be reduced in potential future therapeutic approaches which might reduce the risk of side effects. Interestingly, CsA and FK506 are licensed immunosuppressive drugs applied to patients for example after organ transplantation (Liu et al. 1991). Rad and VER have been tested in anti-tumor treatment which revealed some side effects (Li and Buchner 2013; Schlecht et al. 2013). Besides reduction of concentration and duration of treatment, local application strategies and development of novel inhibitor derivative with improved safety profiles might also help to lower adverse effects.

Moreover, we and others showed that not only clostridial binary toxins but several other toxins depend on chaperones and PPIases (Lang et al. 2014; Ernst et al. 2017b). These toxins are amongst others diphtheria toxin (Schuster et al. 2017), cholera toxin (Burress et al. 2014, 2019; Kellner et al. 2019), and pertussis toxin (Ernst et al. 2018a) which are important virulence factors and the causative agents of severe diseases (Table 1). Comparable to *C. difficile*-associated diseases, therapeutic options for these diseases are limited, and novel approaches based on chaperones and PPIases might provide the possibility of a more universal therapeutic strategy.

## Supplementary information

ESM 1 (PPTX 15912 kb)

ESM 2 (DOCX 13 kb)

## Data Availability

All data generated or analyzed during this study are included in this published article.
