# Supplementary material for: Intoxication of mammalian cells with binary clostridial enterotoxins is inhibited by the combination of pharmacological chaperone inhibitors
Source: Naunyn Schmiedebergs Arch Pharmacol. 2020 Dec 7;394(5):941–54. doi: 10.1007/s00210-020-02029-3 (PMC8102464; doi:10.1007/s00210-020-02029-3)
Supplement: Supplementary file 1 — (PPTX 15912 kb) [file 210_2020_2029_MOESM1_ESM.pptx]

## Slide 1
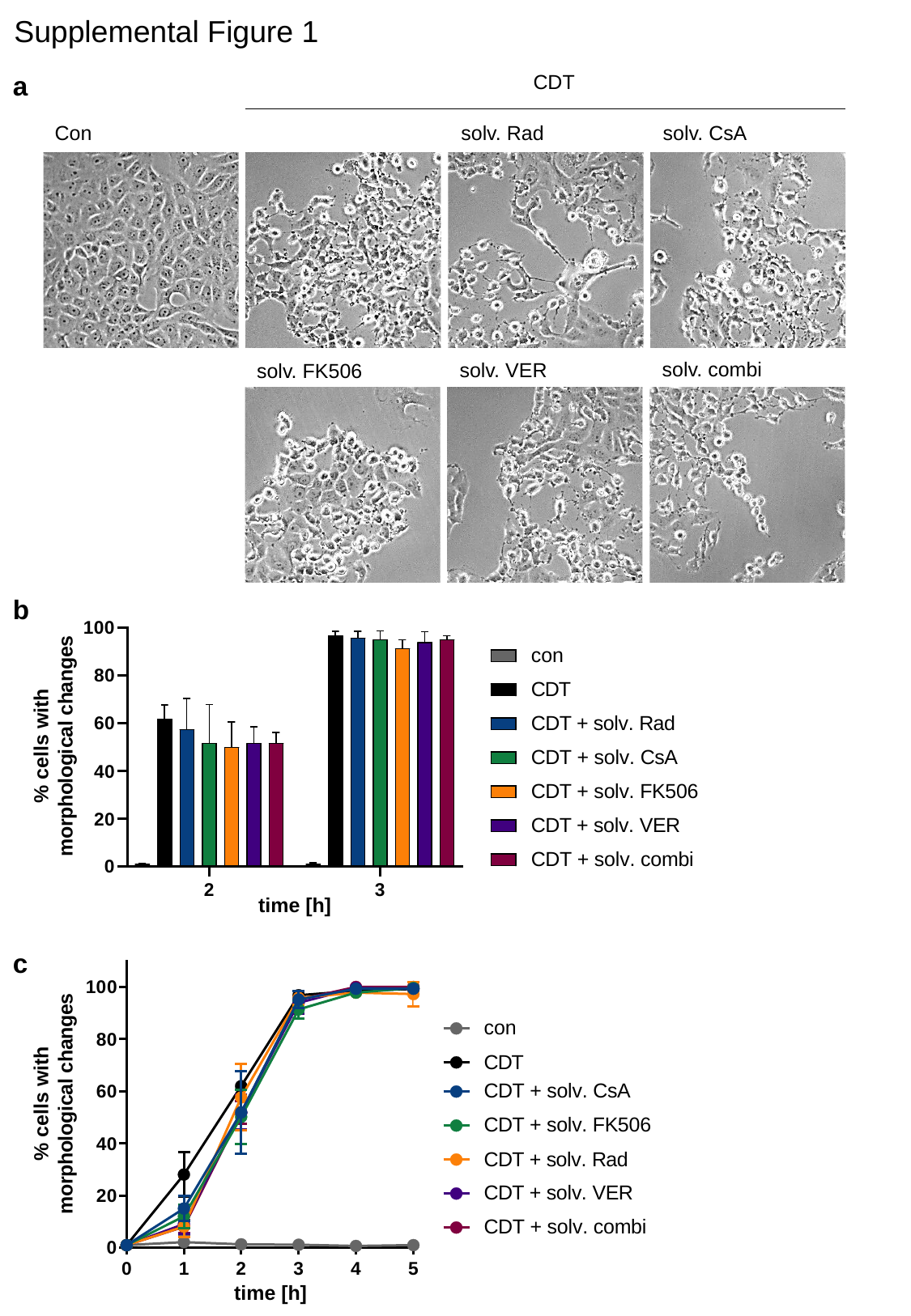

Supplemental Figure 1
a
CDT
solv. CsA
Con
solv. Rad
solv. combi
solv. VER
solv. FK506
b
c

## Slide 2
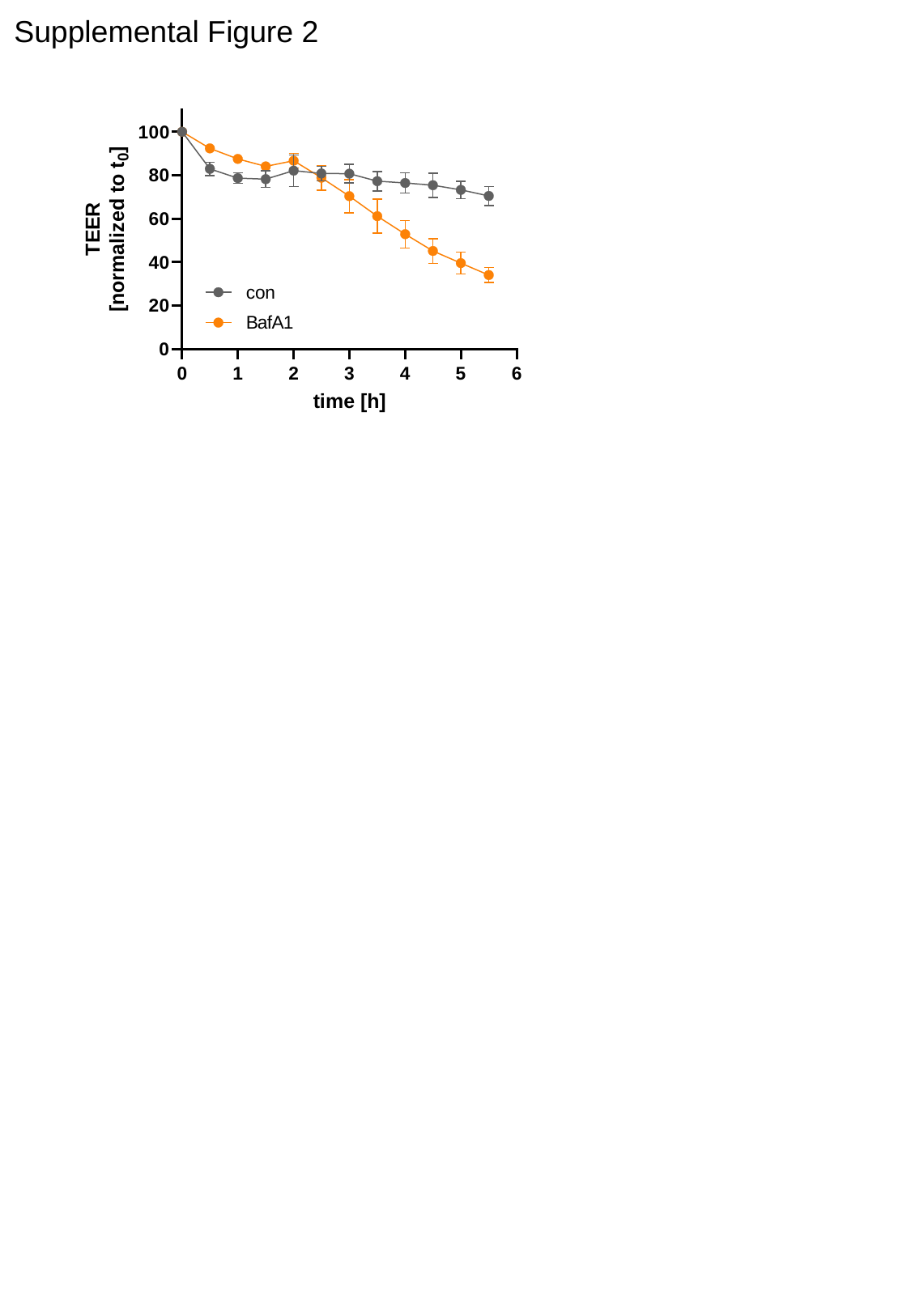

Supplemental Figure 2

## Slide 3
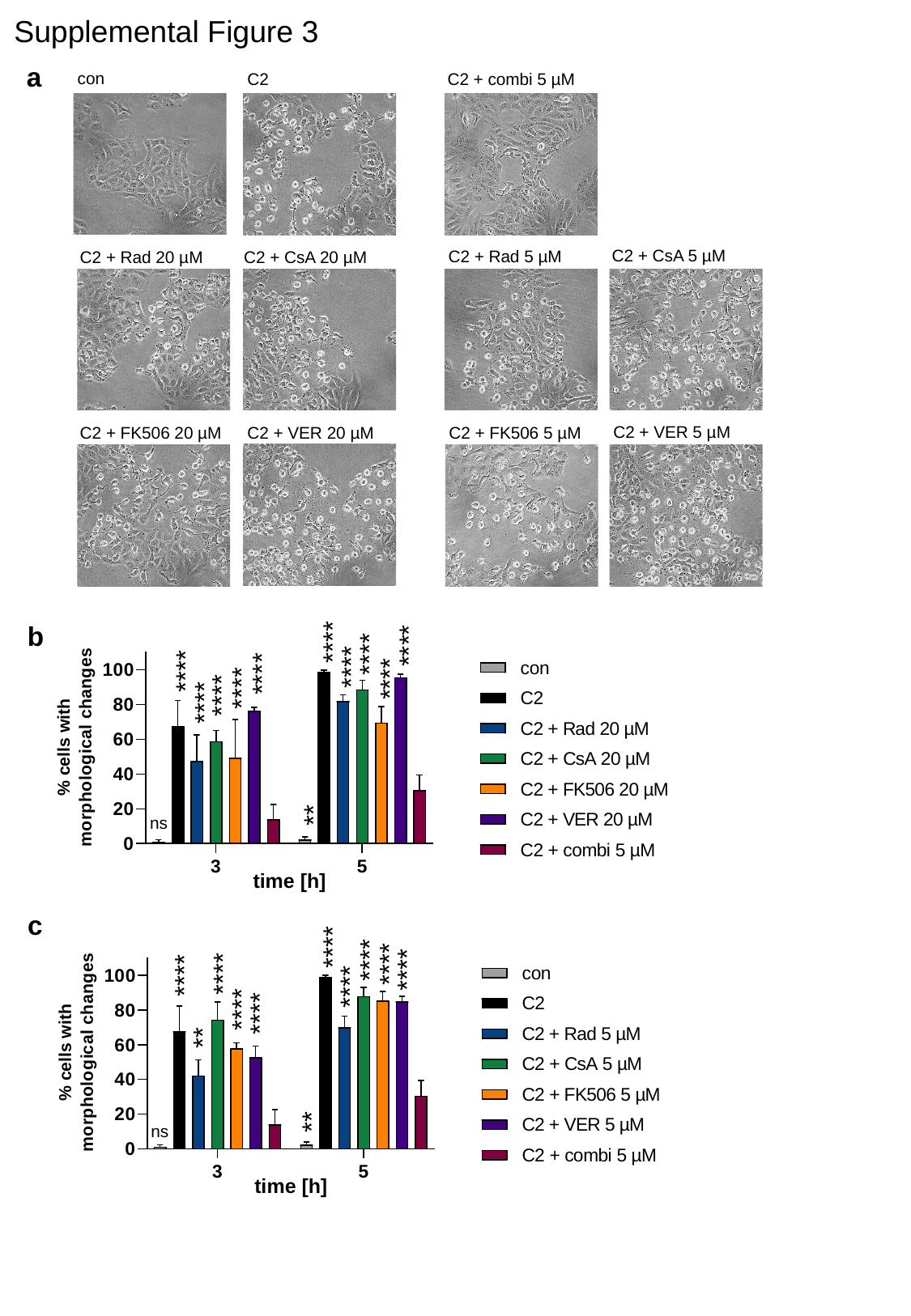

Supplemental Figure 3
a
con
C2
C2 + combi 5 µM
C2 + CsA 5 µM
C2 + Rad 5 µM
C2 + CsA 20 µM
C2 + Rad 20 µM
C2 + VER 5 µM
C2 + FK506 20 µM
C2 + VER 20 µM
C2 + FK506 5 µM
****
****
b
****
****
****
****
****
****
****
****
**
ns
c
****
****
****
****
****
****
****
****
****
**
**
ns

## Slide 4
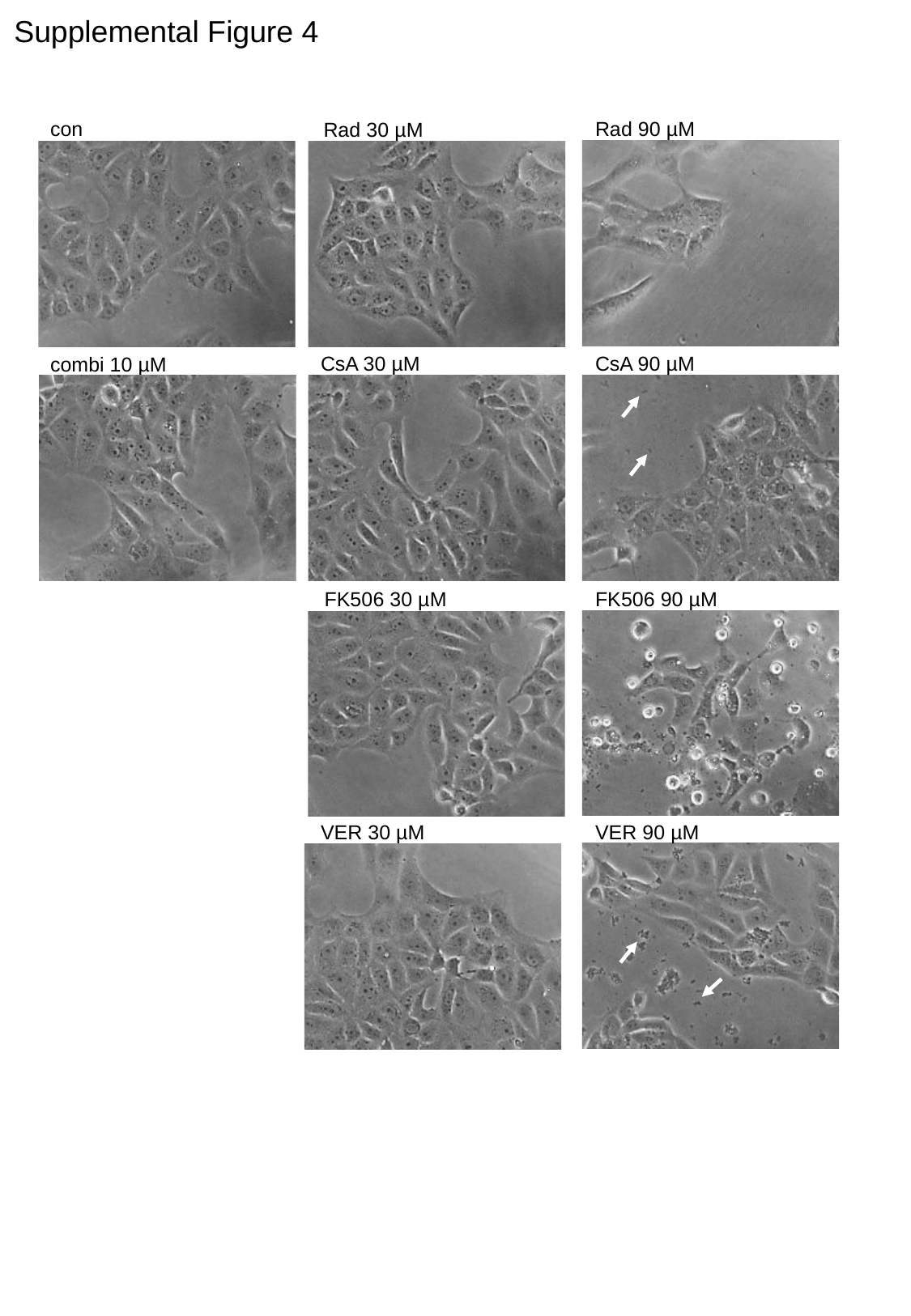

Supplemental Figure 4
Rad 90 µM
con
Rad 30 µM
CsA 90 µM
CsA 30 µM
combi 10 µM
FK506 30 µM
FK506 90 µM
VER 30 µM
VER 90 µM
