# Supplementary material for: Intoxication of mammalian cells with binary clostridial enterotoxins is inhibited by the combination of pharmacological chaperone inhibitors
Source: Naunyn Schmiedebergs Arch Pharmacol. 2020 Dec 7;394(5):941–54. doi: 10.1007/s00210-020-02029-3 (PMC8102464; doi:10.1007/s00210-020-02029-3)
Supplement: Supplementary file 2 — (DOCX 13 kb) [file 210_2020_2029_MOESM2_ESM.docx]

**Intoxication of mammalian cells with binary clostridial enterotoxins is inhibited by the combination of pharmacological chaperone inhibitors**

Katharina Ernst*^#^, Judith Sailer^#^, Maria Braune and Holger Barth*

Institute of Pharmacology and Toxicology, Ulm University Medical Center, 89081 Ulm, Germany

^#^authors contributed equally

*corresponding authors

Katharina Ernst, katharina.ernst@uni-ulm.de, ORCID iD: 0000-0002-9832-9917

Holger Barth, holger.barth@uni-ulm.de, ORCID iD: 0000-0002-2706-3402

**Supplemental Figures**

**Supplemental Fig 1 Solvents of chaperone inhibitors do not affect intoxication of cells with CDT** Vero cells were pre-incubated at 37°C for 30 min with the respective solvents (ethanol or DMSO) of single inhibitors or the combination of inhibitors. The amount of solvent tested corresponds to the amount used in Fig. 1. Cells were then challenged with 50 ng/mL CDTa + 100 ng/mL CDTb. For control, cells were left untreated or treated only with CDT. Cells were further incubated at 37 °C and images were taken at the indicated time points. **(a)** Images show the morphological changes induced by the toxin after 2 h of incubation. **(b)** Percentage of cells with morphological changes was determined from images at the indicated time points. Values are given as mean +/- SD (n = 3). **(c)** Time-course of intoxication with CDT determined from images.

**Supplemental Fig 2 Effect of BafA1 on TEER of CaCo-2 monolayer.** Caco-2 monolayers were treated with the 100 nM BafA1 or left untreated for control. TEER was measured at indicated time points. One representative experiment of three independent experiments is shown. Values were normalized to t_0_ values (t_0_ = 100 %) and given as mean +/- SD (n = 2).

**Supplemental Fig 3 Inhibitor combination protects cells from C2-intoxication in reduced concentrations compared to single inhibitors.** Vero cells were pre-incubated with single inhibitors (Rad, CsA, FK506, VER, 20 µM or 5 µM) or with the combination of inhibitors (5 µM of each inhibitor). C2 toxin was added (50 ng/mL C2I + 100 ng/mL C2IIa) and cell morphology was monitored. **(a)** Cell images are shown exemplarily after 3 h of incubation with C2 toxin. Percentage of cells with morphological changes was determined from cell images. For better visualization, comparison of 20 µM of single inhibitors vs. 5 µM of inhibitor combination are shown in **(b)** and comparison of 5 µM of single inhibitors vs. 5 µM of inhibitor combination are shown in **(c)**. Values for con, C2 and C2 + combi 5 µM are identical in both graphs. Values are given as mean +/- SD (n = 3 images per condition, experiment was repeated independently at least two times). Significance was tested using Two-Way ANOVA followed by Dunnett’s multiple comparison test. (**** p ≤ 0.0001, ** p ≤ 0.01, ns = not significant vs. C2 + combi 5 µM).

**Supplemental Fig 4 Increasing concentrations of chaperone inhibitors cause morphological changes or precipitation of inhibitors.** Vero cells were incubated with Rad, CsA, FK506, VER or the combination of all four inhibitors at indicated concentrations. Cell images were taken after 6 h before the medium was changed and cell viability was measured (results shown in Fig. 5d). One representative cell image is shown. 3 images were taken per treatment and the experiment was performed independently at least three times. Arrows indicate precipitation.
